# The RIFLE versus AKIN classification for incidence and mortality of acute kidney injury in critical ill patients: A meta-analysis

**DOI:** 10.1038/srep17917

**Published:** 2015-12-07

**Authors:** Jiachuan Xiong, Xi Tang, Zhangxue Hu, Ling Nie, Yiqin Wang, Jinghong Zhao

**Affiliations:** 1 Department of Nephrology, Institute of Nephrology of Chongqing and Kidney Center of PLA, Xinqiao Hospital, Third Military Medical University, Chongqing, 400037, China; 2 Department of Nephrology, West China Hospital, Sichuan University, Chengdu 610041, China

## Abstract

The sensitivity and accuracy of the Risk/Injury/Failure/Loss/End-stage (RIFLE) versus acute kidney injury Network (AKIN) criteria for acute kidney injury (AKI) in critically ill patients remains uncertain. Therefore, we performed a systematic review and meta-analysis to investigate the incidence and prognostic value of the RIFLE versus AKIN criteria for AKI in critically ill patients. Literatures were identified by searching Medline, Embase, PubMed, and China National Knowledge Infrastructure (CNKI) database. Nineteen studies with 171,889 participants were included. The pooled estimates of relative risk (RR) were analyzed. We found that the RIFLE and AKIN criteria is different for the incidence of AKI in intensive care unit (ICU) patients (P = 0.02, RR = 0.88), while not for cardiac surgery patients (P = 0.30, RR = 0.93). For AKI-related hospital mortality, the AKIN criteria did not show a better ability in predicting hospital mortality in either ICU (P = 0.19, RR = 1.01) or cardiac surgery patients (P = 0.61, RR = 0.98) compared to RIFLE criteria. Our findings supported that the AKIN criteria can identify more patients in classifying AKI compared to RIFLE criteria, but not showing a better ability in predicting hospital mortality. Moreover, both RIFLE and AKIN criteria for AKI in cardiac surgery patients had better predictive ability compared with the ICU patients.

Acute kidney injury (AKI) is the newly name replaced the term acute renal failure^1^. It is manifested with changes in urine output, creatinine and blood chemistries^2^. AKI is common worldwide and is associated with significant morbidity, mortality, and resource use with a higher risk for the development of chronic kidney disease (CKD) especially for critical ill patients^3^,^4^,^5^,^6^. Using the Kidney Disease: Improving Global Outcome (KDIGO) definition, a meta-analysis showed that the incidence of AKI in adults and children were 21.6% and 33.7%, respectively, the AKI-associated mortality rates were 23.9% and 13.8% in adults and children, respectively^7^. AKI is a common and important diagnostic and therapeutic challenge for clinicians^8^. More than 200 different definitions of AKI were provided^9^. These multiple definitions make for clinical confusion and difficulty in the diagnosing of this condition^10^. Several classifications for AKI have been made during the past few years to better define this disease. A consensus definition of AKI was published by the Acute Dialysis Quality Initiative (ADQI) in 2004^1^. This consensus definition is termed the Risk/Injury/Failure/Loss/End-stage (RIFLE) criteria, and the following categories were used: ‘Risk’ is the least severe category of AKI, followed by ‘Injury’, ‘Failure’, ‘Loss’ and ‘End-stage renal disease’. In 2007, a modified version of the RIFLE criteria was published by the AKI Network (AKIN)—known as the AKIN criteria^11^. Definition of AKI: the categories of Risk, Injury, and Failure. Since then, many studies compared the two measures to evaluate the incidence and risk factor of AKI, some studies evaluated the sensitivity and accuracy of the RIFLE and AKIN criteria for critically ill patients, some authors also proposed to evaluate the hospital mortality and outcome of AKI patients by the two classifications. However, the results are still inconclusive. In the present study, we aimed to perform a systematic review and pool the available data to evaluate the incidence and prognostic value of the RIFLE and AKIN classification for AKI patients.

## Results

### Flow and Study Characteristics

**Figure 1:**
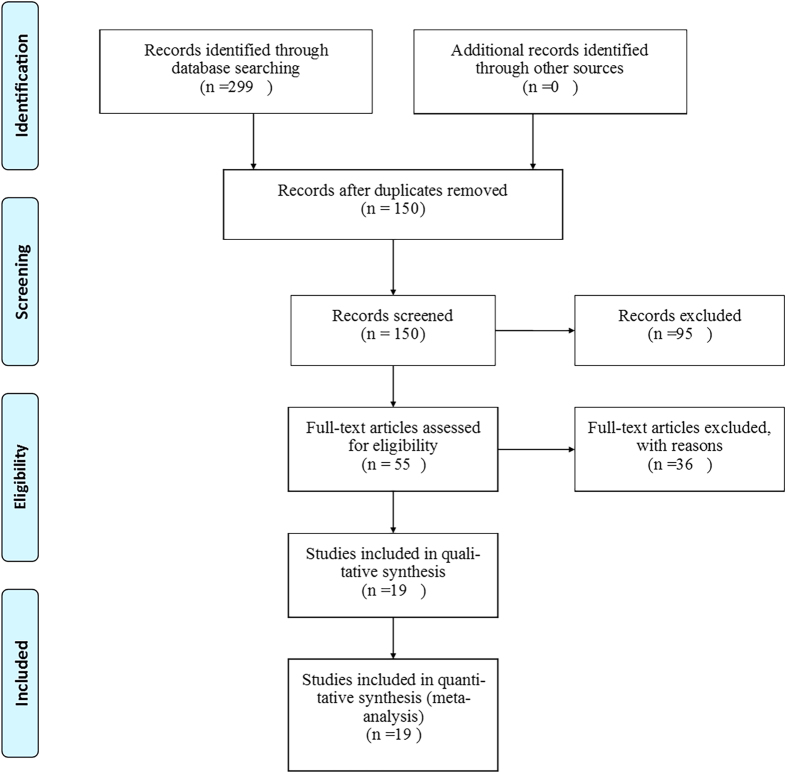
Flow diagram of included/excluded studies.

The literature search yielded 299 articles, 19 studies^12^,^13^,^14^,^15^,^16^,^17^,^18^,^19^,^20^,^21^,^22^,^23^,^24^,^25^,^26^,^27^,^28^,^29^,^30^ were eligible for inclusion, and flow diagram of included/excluded studies was showed in Fig. 1. Twelve studies for Intensive care units (ICU) with 138,521 patients, 7 studies for cardiac surgery with 33,038 patients. There are 8 studies from Asia, 5 from Europe, both America and Australia have 2 studies, each of Brazil and Bitlis had one study. 15 studies were retrospective, and 4 studies were prospective, all included studies are compare RIFLE with AKIN for AKI patients in ICU or cardiac surgery, the basic characteristics of the included studies are summarized in Table 1. Among these studies, 7 studies were multicenter, 12 studies were from single center, 14 studies use hospital mortality to evaluate mortality endpoint, 1 study uses mortality of ARF, and 1 study with 28-day mortality, 3 studies did not acquire the endpoint information. The quality of the including studies by QUDAS II was listed below (Supplementary figure 1).

### The RIFLE VS. AKIN classification for the incidence of AKI in ICU patients

**Figure 2:**
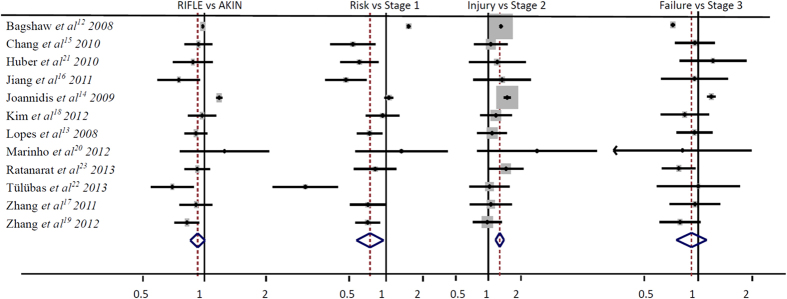
The RIFLE VS. AKIN classification for the incidence of AKI in ICU patients.

Twelve studies with 138,521 patients were included for the incidence of AKI in ICU patients, 50,054 patients were diagnosed AKI with RIFLE classification and 50,521 patients were diagnosed AKI with AKIN classification. As shown in Tables 2 and 3, the incidence of AKI range from 18.13%–66.67% and 24.14%–76.67% by RIFLE and AKIN classification, respectively. The total incidence of AKI diagnosed by RIFLE and AKIN classification showed a significance difference (RR, 0.88; 95%CI, 0.80–0.98; P = 0.02; Fig. 2), for each stage, the Risk VS. Stage 1 (RR, 0.70; 95%CI, 0.53–0.93; P = 0.02; Fig. 2) and the Injury VS. Stage 2 (RR, 1.29; 95%CI, 1.17–1.43; P < 0.00001; Fig. 2) for the incidence of AKI were showed a significant difference, but the Failure VS. Stage 3 (RR, 0.90; 95%CI, 0.73–1.11; P = 0.34; Fig. 2) for the incidence of AKI in ICU patients were not showed significant difference.

### The RIFLE VS. AKIN classification for the hospital mortality of AKI in ICU patients

**Figure 3:**
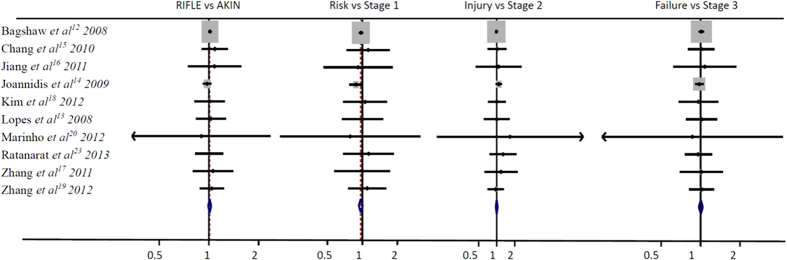
The RIFLE VS. AKIN classification for the hospital mortality of AKI in ICU patients.

There are 10 studies to evaluate the mortality for AKI patients, 49,879 patients diagnosed with AKI using the RIFLE classification and 13,423 patients died in hospital, 13,279 patients died in hospital with a total 50,275 AKI patients using AKIN classification, As shown in Tables 2 and 3, the total hospital mortality was 0.27 and 0.26 by RIFLE and AKIN classification respectively, both classifications were not showed statistical significance (RR, 1.01; 95%CI, 0.99–1.03; P = 0.19; Fig. 3), for each stage, the Risk VS. Stage 1 (RR, 0.96; 95%CI, 0.93–1.00; P = 0.04; Fig. 3) was showed a significant difference, but both the Injury VS. Stage 2 (RR, 1.00; 95%CI, 0.96–1.03; P = 0.95; Fig. 3) and the Failure VS. Stage 3 (RR, 1.01; 95%CI, 0.92–1.04; P = 0.75; Fig. 3) for the hospital mortality of AKI were not showed a significant difference.

### The RIFLE VS. AKIN classification for AKI in cardiac surgery patients

**Figure 4:**
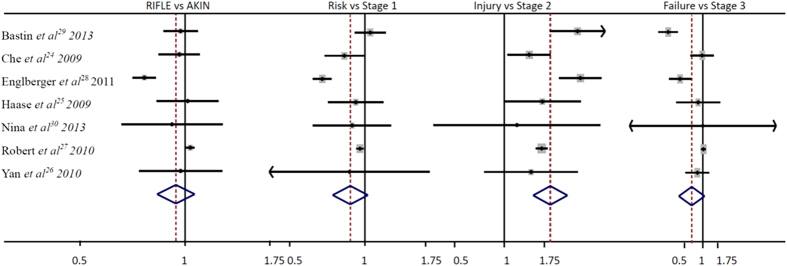
The RIFLE VS. AKIN classification for AKI in Cardiac surgery patients.

Seven studies with 33,038 patients reported the RIFLE and AKIN classification for AKI in cardiac surgery patients, AKI were 9,659 patients with RIFLE classification and 9,717 patients with AKIN classification. The information was listed in Tables 3 and 4, and the two classifications did not have a difference to diagnosis the incidence of AKI (RR, 0.93; 95%CI, 0.81–1.07; P = 0.30; Fig. 4), for each stage comparison, the Risk VS. Stage 1 (RR, 0.85; 95%CI, 0.71–1.03; P = 0.09; Fig. 4) and the Failure VS. Stage 3 (RR, 0.64; 95%CI, 0.39–1.05; P = 0.08; Fig. 4) for the incidence of AKI were also not showed a statistical significant difference, but the Injury VS. Stage 2 (RR, 2.06; 95%CI, 1.61–2.64; P < 0.00001; Fig. 4) was quiet different with the incidence of AKI in cardiac surgery patients.

### The RIFLE VS. AKIN classification for the hospital mortality of AKI in cardiac surgery patients

**Figure 5:**
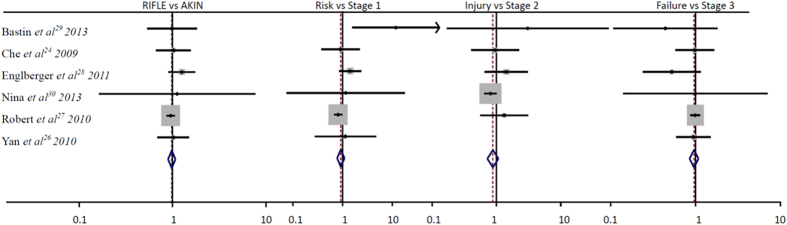
The RIFLE VS. AKIN classification for the hospital mortality of AKI in cardiac surgery patients.

Six studies to evaluate the mortality for AKI in cardiac surgery patients, 9,530 patients diagnosed with AKI using the RIFLE classification and 821 patients died in hospital, 837 patients died in hospital with a total of 9,591 AKI patients using AKIN classification. As showed in Tables 3 and 4, the total hospital mortality in cardiac surgery patients by RIFLE and AKIN classification were not showed significant difference, the RR ratio were 0.98, 95%CI, 0.89–1.07; P = 0.61; Fig. 5, for each stage comparison, there were also not showed a statistical significant difference among the Risk VS. Stage 1(RR, 1.16; 95%CI, 0.71–1.89; P = 0.55; Fig. 5), the Injury VS. Stage 2(RR, 0.87; 95%CI, 0.71–1.06; P = 0.16; Fig. 5) and the Failure VS. Stage 3(RR, 0.92; 95%CI, 0.78–1.09; P = 0.34; Fig. 5).

### Area under the receiver operator characteristics (AuROC) curves for the RIFLE and AKIN classification for hospital mortality of AKI

**Figure 6:**
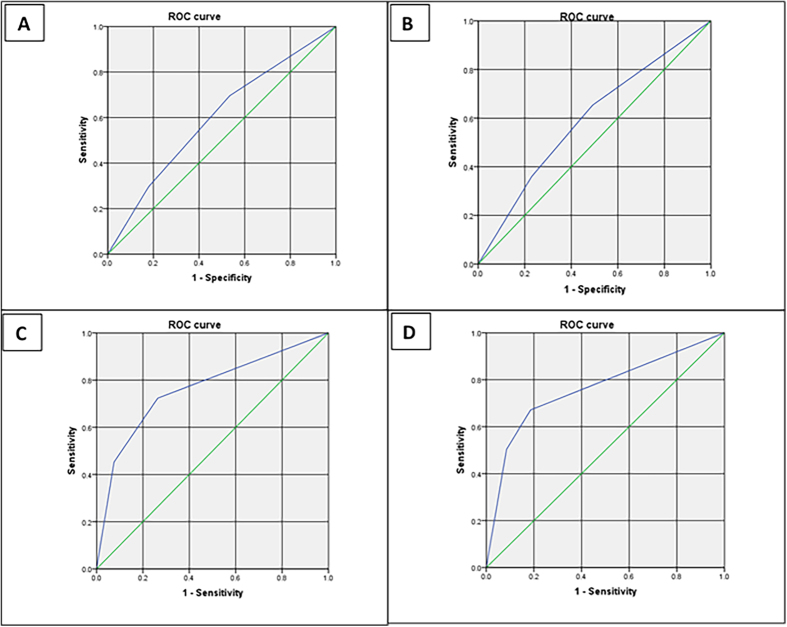
Area under the receiver operator characteristics (AuROC) curves for the RIFLE and AKIN classification. (**A**) The RIFLE classification for the incidence of AKI in ICU patients (AuROC = 0.598). (**B**) The AKIN classification for the incidence of AKI in ICU patients (AuROC = 0.594). (**C**) The RIFLE classification for the hospital mortality of AKI in cardiac surgery patients (AuROC = 0.762). (**D**) The AKIN classification for the hospital mortality of AKI in cardiac surgery patients (AuROC = 0.761).

The AuROC curve for incidence was 0.598 for RIFLE classification (95%CI, 0.592–0.603, P<0.0001) and was 0.594 for AKIN classification (95%CI, 0.589–0.600, P < 0.0001) for hospital mortality of AKI in ICU patients, whereas the AuROC curve was 0.762 (95%CI, 0.743–0.782, P < 0.0001) for RIFLE classification and was 0.761 for AKIN classification (95%CI, 0.741–0.781, P < 0.0001) for hospital mortality of AKI in cardiac surgery patients, although the AuROC curve was not significant between RIFLE and AKIN classification in either ICU patients or cardiac surgery patients, the AuROC of both RIFLE and AKIN classification for cardiac surgery patients had better predictive ability compared with the ICU patients (Fig. 6).

### Publication bias

Begg’s funnel plot was performed to access the publication bias of the literature. The shapes of the funnel plots revealed some evidence of obvious asymmetry. The funnel plot of the incidence of AKI in ICU patients and the incidence of AKI in cardiac patients were showed in Supplementary Figure 2 and 3, respectively.

## Discussion

Acute kidney injury is very common with high hospital mortality in critically ill patients^31^, epidemiological studies demonstrate the wide variation in etiologies and risk factors, developing into chronic kidney disease and progression to dialysis dependency^32^,^33^. However, there is lack of a universally accepted and standardized definition for AKI for nephrologists and health care workers. The Acute Dialysis Quality Initiative’s RIFLE criteria has been validated in several clinical settings and shown to correlate with important outcomes, such as needing for renal replacement therapy (RRT), length of hospital stay, and mortality^22^,^34^,^35^,^36^. But it is still imperfect. In 2007, the AKIN convened to refine the RIFLE criteria. A few modifications were added to AKI including the eliminating the change of glomerular filtration rate (GFR) and the outcome categories of Loss and ESRD; the stage 1 was redefined with an absolute increase in creatinine of at least 0.3 mg/dl; patients starting RRT are automatically classified as stage 3. Both the RIFLE and AKIN classifications use the changes of serum creatinine or urine output to establish the clinical syndrome of AKI in 3 severity levels. But whether the sensitivity of AKIN in the diagnosis of AKI in ICU patients has clinical significance, or the new classification can representative the severity of AKI patients and good predictive value for prognosis is not very clear.

A number of epidemiologic studies have tried to compare the RIFLE and AKIN criteria for the incidence and in-hospital mortality of AKI in critical ill patients and cardiac surgery patients^12^,^13^,^14^,^25^,^27^,^28^. Bagshaw and their college work revealed that the AKIN criteria do not improve the sensitivity and predictive ability of classification of AKI in the first 24 h after admission to ICU compared to the RIFLE criteria^12^. However, Ratanarat *et al.*^23^ found that AKIN criteria improved sensitivity for detection of AKI and prediction of in-hospital mortality was better than that of RIFLE criteria in critically ill patients with multi-organ dysfunction syndrome, Lopes *et al.*^13^, Zhang *et al.*^17^ and Jiang *et al.*^16^ showed that although AKIN criteria improved sensitivity of AKI diagnosis but does not improve ability in predicting in-hospital mortality of critically ill patients. In cardiac surgery patients, Haase *et al.*^25^ reported that the AKIN classification do not materially improve the clinical usefulness of RIFLE definition, however, Yan *et al.*^26^ found that the AKIN criteria seem not to have greater sensitivity and specificity compared with the RIFLE classification.

In the present study, 19 studies with more than 171,559 participants are included in our meta-analysis. We have consistently confirmed that patients with AKI, the RIFLE and AKIN classification is different for the incidence of AKI in ICU patients, the incidence of AKI diagnosed by AKIN classification is higher than the RIFLE classification, in subgroup analysis, we found that Risk vs. Stage 1 and Injury vs. Stage 2 were also different, but the Failure vs. Stage 3 did not show a statistical significance. This may be explained by the change criteria in stage 1 and the elimination of GFR, thus more patients were diagnosed as AKI by AKIN classification, and there still some patients needs RRT for fluid overload were stratified in Stage 3 without increased serum creatinine which cannot be included in Failure stage by RIFLE. All this contributes to the high incidence of AKI by AKIN criteria. In cardiac surgery patients with AKI, the RIFLE and AKIN classification did not show difference for the diagnosis, only Injury vs. Stage 2 has a significant difference by subgroup analysis while the other stages did not. Comparing to cardiac surgery patients, the AKI incidence is higher in ICU patients, although some cardiac surgery patients were transferred to ICU. ICU is mixed with all kind of critical ill patients such as septic shock, acute respiratory distress syndrome, or hepatic cirrhosis. We also evaluated the in-hospital mortality, and found that both RIFLE and AKIN criteria did not reach a significant difference to predict mortality, by subgroup analysis, only Risk VS. Stage 1 of AKI in ICU patients reached a significant difference. Thus, from our pooled analysis, our findings support the RIFLE or AKIN definition for AKI in ICU patients is different, but both classifications were not different for mortality. But we did find that both RIFLE and AKIN classification had a better predictive ability for cardiac surgery patients compared with ICU patients, which means that the predictive value for AKI related in-hospital mortality may different according to the different patients. This needs other patients to confirm our results.

In 2012, the Kidney Disease Improving Global Outcomes (KDIGO) Work Group proposed another definition that builds upon the AKIN definition, attempting to harmonize earlier consensus definitions and staging criteria for AKI. But Palevsky *et al.* thought that the KDIGO definition and staging criteria are appropriate for defining the epidemiology of AKI and is insufficient evidence to support their widespread application to clinical care in the United States^37^. A retrospective cohort study of 31,970 hospitalizations performed by Zeng *et al.*^38^ showed that AKI incidence was highest according to the KDIGO definition (18.3%) followed by the AKIN (16.6%), and RIFLE (16.1%). In addition, another retrospective observational study of 49,518 admissions indicated that 11.6% were diagnosed with KDIGO criteria, 11.0% were diagnosed with RIFLE criteria, and only 4.8% were diagnosed with AKIN criteria^39^. In critically ill patients, KDIGO was more predictive than the RIFLE criteria, but there was no significant difference between AKIN and KDIGO^40^, Luo *et al.* found that the incidence of AKI using the RIFLE, AKIN, and KDIGO criteria were 46.9%, 38.4%, and 51%, respectively^40^. Moreover, for in-hospital mortality, only small differences in predictive abilities between RIFLE and KDIGO concerning clinical outcomes at 30 days in acute decompensated heart failure patients^41^. Therefore, all these definitions have their own limitation for wide accepted, new classification was needed to establish an early diagnosis of AKI that would be a simple and useful clinical tool.

The major limitation is that most of the included studies were retrospective, and the AKI incidence was a large range in different medical center, which cause a heterogeneity. Second, some studies were multicenter which increased the weight in the meta-analysis. Third, we only evaluate the AKI in ICU and cardiac patients, the other diseases or syndromes associated acute kidney injury were not included in our meta-analysis. At last, A publication bias may have occurred. The funnel plot shows significant evidence of the bias (Supplementary Figures 2 and 3).

In conclusion, our study found that the AKIN criteria can identify more patients in classifying AKI in ICU patients compared to RIFLE criteria but not cardiac patients, for the prediction of AKI-related mortality, the AKIN criteria did not show a better ability in predicting hospital mortality in both ICU and cardiac surgery patients compared to RIFLE criteria. But both the RIFLE and AKIN classifications for AKI in cardiac surgery patients had better predictive ability compared with the ICU patients.

## Materials and Methods

### Data Sources, Search Strategy, and Selection Criteria

We performed a systematic search to identify the studies examined the RIFLE and AKIN criteria for acute kidney injury. Literatures were identified by searching MEDLINE via Ovid, EMBASE, PubMed, and China National Knowledge Infrastructure (CNKI) database. The last updated search was performed on November 1st, 2013. The searching terms were “Risk or Injury or Failure or RIFLE”, “AKIN or Acute Kidney Injury Network” and “acute renal failure or acute kidney injury or AKI”. We manually searched the references of the identified studies and review articles, and academic congresses on kidney disease with available data were also included. The search was limited to compare the RIFLE and AKIN classification for AKI in ICU and cardiac surgery patients out without restriction on language.

### Data extraction

Two authors independently extracted the information from all eligible publications using standard data extraction forms. Disagreement was resolved by discussion between the two authors, or the consultation with a third reviewer. The standardized data form was used for data collection, including first author, year of publication, country of origin, ethnicity of the study. The data of baseline serum creatinine, length of stay and renal replace therapy were recorded when available. All completed studies that compare AKI was defined and classified by the RIFLE criteria and the AKIN criteria in ICU or cardiac patients were eligible for inclusion.

### Inclusion and Exclusion Criteria

The included studies met the following criteria: (1) Compare the AKIN classification with the RIFLE classification for acute kidney injury; (2) Age ≥16 years; (3) Patients were from ICU or cardiac surgery. Exclusion Criteria: (1) Age <16 years; (2) Length of stay less than 24 hours; (3) Patients with end-stage renal disease (ESRD) on chronic dialysis, prior ESRD or kidney transplant patients readmitted in hospital; (4) Renal allograft patients; (5) if serum creatinine data were not available either during the pre-specified time windows for AKI; (6) Data were unavailable for estimating odds ratio (OR) with 95% confidence interval (CI).

### Evaluations of statistical associations

To summarize the incidence and hospital mortality of AKI patients diagnosed by the RIFLE criteria and AKIN criteria in published studies in adults, we looked at the pooled estimate of relative ratios (RRs) and 95% confidence intervals (CIs) were calculated for AKI patients with the two classifications. We also looked at the RRs comparing the three RIFLE classes Risk, Injury, and Failure with AKIN stages (Risk VS. Stage 1, Injury VS. Stage 2 and Failure VS. Stage 3). Data were combined using a random effects model or fixed effects model according to the I^2^ test, I^2^ value 25%, 50% and 75% correspond to low, medium and high levels of heterogeneity, Fixed effects model was selected while the I^2^ under 50%, otherwise the a random effects model will be used^42^. Analysis was performed with Stata software, version 12. We used QUDAS II to evaluate the quality of the including studies and Asymmetry funnel plots were used to assess potential publication bias by Revman software, version 5.3. Model fit was assessed by the goodness of-fit test, and discrimination was assessed by the area under the receiver operator characteristic (AuROC) curve (SPSS version 20). The p value less than 0.05 was considered as a statistical significance.

## Additional Information

**How to cite this article**: Xiong, J. *et al.* The RIFLE versus AKIN classification for incidence and mortality of acute kidney injury in critical ill patients: A meta-analysis. *Sci. Rep.* **5**, 17917; doi: 10.1038/srep17917 (2015).

## Supplementary Material

Supplementary Information

## Figures and Tables

**Table 1:** Summary of original investigations using the RIFLE and AKIN classification for AKI.

Author	Year	Country	N, total	Age (y)	M:F	Baseline serum creatinine(μmol/l)	Length of stay (days)	RRT	Population studied	Retrospective/prospective	Single/multicenter	Mortality end point
Bagshaw^12^	2008	Australia	120123	61.6(44–79)	1.5:1	98	NA	NA	ICU	Retrospective	Multicenter	Hospital mortality
Lopes^13^	2008	Portugal	662	58.6(39–78)	1.5:1	86	7	NA	ICU	Retrospective	Single	Hospital mortality
Joannidis^14^	2009	Australia	14365	63 (49–74)	1.6:1	NA	NA	NA	ICU	Retrospective	Multicenter	Hospital mortality
Chang^15^	2010	China	291	62	2.3:1	194	11.4	NA	ICU	Retrospective	Multicenter	Hospital mortality
Jiang^16^	2011	China	524	56.5	2.1:1	94.9	7	28	ICU	Retrospective	Single	Hospital mortality
Zhang^17^	2011	China	331	60	1.4:1	94	9.1	NA	ICU	Retrospective	Single	Hospital mortality
Kim^18^	2012	Korea	291	50.5	2.1:1	175	NA	97	ICU	Retrospective	Single	28-day mortality
Zhang^19^	2012	China	1036	51.1(37–66)	1.6:1	98.7	6.1	75	ICU	Retrospective	Single	Hospital mortality
Marinho^20^	2012	Portugal	87	67.6	1.4:1	NA	19.6	NA	ICU	Prospective	Multicenter	NA
Huber^21^	2012	Germany	321	62.2	1.2:1	151	9.1	16	ICU	Retrospective	Single	NA
Tülübaş^22^	2013	Bitlis	190	45.43(18–70)	1.6:1	NA	NA	NA	ICU	Prospective	Single	Mortality of ARF
Ratanarat^23^	2013	Thailand	300	60(43–74)	1.1:1	79	17	102	ICU	Retrospective	Single	Hospital mortality
Che^24^	2009	China	1056	57.3	1.4:1	77.4	NA	19	Cardiac surgery	Retrospective	Single	Hospital mortality
Haase^25^	2009	Germany	282	NA	NA	NA	NA	NA	Cardiac surgery	Prospective	Single	NA
Yan^26^	2010	China	67	61.1	2.6:1	78.1	NA	30	Cardiac surgery	Retrospective	Single	Hospital mortality
Robert^27^	2010	USA	24747	66	2.4:1	97	NA	NA	Cardiac surgery	Prospective	Multicenter	Hospital mortality
Englberger^28^	2011	USA	4836	67(18–100)	1.9:1	100	6	96	Cardiac surgery	Retrospective	Multicenter	Hospital mortality
Bastin^29^	2013	UK	1881	66 (56–74)	2.5:1	98	NA	18	Cardiac surgery	Retrospective	Single	Hospital mortality
Nina^30^	2013	Brazil	169	63.4	2:1	NA	NA	NA	Cardiac surgery	Retrospective	Single	Hospital mortality

N, patients number; y, year; M:F, male to female ratio; RRT, renal replacement therapy; ICU, intensive care unit; NA, not acquired.

**Table 2:** RIFLE VS. AKIN classification for the incidence of AKI in ICU Patients and hospital mortality.

Study	Year	RIFLE(No. of death/diagnosis)				AKIN(No. of death/diagnosis)				Incidence of AKI	
		Risk	Injury	Failure	Total	Stage I	Stage II	Stage II	Total	Total	(RIFLE/AKIN)%
Bagshaw^12^	2008	3499/19547	4527/16344	2491/7504	10817/43395	4022/21741	3417/12160	3473/10652	10912/44553	120123	36.13%/37.09%
Lopes^13^	2008	30/97	24/73	66/120	120/290	43/140	22/67	68/127	133/334	662	43.81%/50.45%
Joannidis^14^	2009	319/1092	515/1596	1024/2405	1858/5093	372/1077	300/1033	871/1983	1543/4093	14365	35.45%/28.49%
Chang^15^	2010	24/38	36/52	75/87	135/177	30/57	33/49	78/92	131/198	291	60.82%/68.04%
Jiang^16^	2011	9/34	9/23	18/38	36/95	23/78	6/17	17/40	46/135	524	18.13%/25.76%
Zhang^17^	2011	16/49	16/36	34/60	66/145	23/70	12/34	35/63	70/167	331	43.81%/50.45%
Kim^18^	2012	28/61	44/63	35/59	107/183	28/66	36/52	46/73	110/191	291	62.89%/65.64%
Zhang^19^	2012	36/109	46/75	76/89	158/273	47/161	50/77	95/115	192/353	1036	26.35%/34.07%
Marinho^20^	2012	3/11	2/9	2/8	7/28	3/8	0/3	3/10	6/21	87	32.18%/24.14%
Huber^21^	2012	NA/40	NA/22	NA/42	NA/104	NA/72	NA/18	NA/34	NA/124	321	32.40%/38.63%
Tülübaş^22^	2013	NA/11	NA/36	NA/24	NA/71	NA/63	NA/35	NA/24	NA/122	190	37.37%/64.21%
Ratanarat^23^	2013	20/38	33/62	66/100	119/200	21/48	15/40	100/142	136/230	300	66.67%/76.67%

RIFLE, Risk, Injury, Failure, Loss of kidney function, and End-stage kidney disease; AKIN, acute kidney injury network; AKI, acute kidney injury; NO, patients number; NA, not acquired.

**Table 3:** The Meta-analysis performed on different settings.

Compared AKI levels	AKI in ICU patients				Hospital mortality in ICU patients			
	Incidence(RIFLE/AKIN)	RR (95% CI)	P(overall effect)	P (heterogeneity)	Mortality(RIFLE/AKIN)	RR (95% CI)	P(overall effect)	P (heterogeneity)
RIFLE VS. AKIN	0.36/0.36	0.88[0,80,0.98]	0.02	<0.00001	0.27/0.26	1.01[0.99,1.03]	0.19	0.53
Risk VS. Stage 1	0.15/0.17	0.70[0.60,0.81]	<0.00001	<0.00001	0.19/0.20	0.96[0.93,1.00]	0.04	0.51
Injury VS. Stage 2	0.13/0.10	1.29[1.17,1.43]	<0.00001	0.007	0.29/0.29	1.00[0.96,1.03]	0.95	0.65
Failure VS. Stage 3	0.08/0.09	0.90[0.73,1.11]	0.34	<0.00001	0.37/0.36	1.01[0.97,1.04]	0.75	0.97
	AKI in Cardiac surgery patients				Hospital mortality in Cardiac surgery patients			
RIFLE VS. AKIN	0.29/0.29	0.93[0.81,1.07]	0.30	<0.00001	0.09/0.09	0.98[0.89,1.07]	0.61	0.70
Risk VS. Stage 1	0.20/0.23	0.85[0.71,1.03]	0.09	<0.00001	0.03/0.04	1.16[0.71,1.89]	0.55	0.05
Injury VS. Stage 2	0.06/0.03	1.97[1.55,2.49]	<0.00001	0.003	0.12/0.14	0.87[0.71,1.06]	0.16	0.22
Failure VS. Stage 3	0.03/0.03	0.64[0.39,1.05]	0.08	<0.00001	0.36/0.36	0.95[0.85,1.06]	0.33	0.31

RIFLE, Risk, Injury, Failure, Loss of kidney function, and End-stage kidney disease; AKIN, acute kidney injury network; AKI, acute kidney injury; ICU, intensive care unit ; RR, relative risk. CI, confidence interval; P, P value.

**Table 4:** RIFLE VS. AKIN classification for the incidence of AKI in cardiac surgery patients and hospital mortality.

Study	Year	RIFLE(No. of death/diagnosis)				AKIN(No. of death/diagnosis)					incidence of AKI
		Risk	Injury	Failure	Total	Stage I	Stage II	Stage II	Total	Total	(RIFLE/AKIN)%
Che^24^	2009	8/178	11/96	18/38	37/312	11/223	8/66	19/39	38/328	1056	29.55%/31.06%
Haase^25^	2009	NA/85	NA/34	NA/10	NA/129	NA/95	NA/19	NA/12	NA/126	282	45.74%/44.68%
Yan^26^	2010	3/11	12/19	17/24	32/54	3/13	5/12	25/32	33/57	67	80.60%/85.07%
Robert^27^	2010	175/5357	164/1473	328/900	667/7730	229/5659	121/852	324/880	674/7391	24747	31.24%/29.87%
Englberger^28^	2011	27/715	31/169	6/31	64/915	30/1141	7/57	33/74	70/1272	4836	18.92%/26.30%
Bastin^29^	2013	13/336	4/98	2/35	19/469	1/317	0/34	19/136	20/487	1881	24.93%/25.89%
Nina^30^	2013	1/43	0/6	1/1	2/50	1/50	0/5	1/1	2/56	169	29.59%/33.14%

RIFLE, Risk, Injury, Failure, Loss of kidney function, and End-stage kidney disease; AKIN, acute kidney injury network; AKI, acute kidney injury; NO, patients number; NA, not acquired.
