# Supplementary material for: The RIFLE versus AKIN classification for incidence and mortality of acute kidney injury in critical ill patients: A meta-analysis
Source: Sci Rep. 2015 Dec 7;5:17917. doi: 10.1038/srep17917 (PMC4671151; doi:10.1038/srep17917)
Supplement: Supplementary Information [file srep17917-s1.doc]

**The RIFLE versus AKIN classification for incidence and mortality of acute kidney injury in critical ill patients：A systematic review and meta-analysis**

Jiachuan Xiong^1^，Xi Tang^2^，Zhangxue Hu^2^，Ling Nie^1^，Yiqin Wang^1^，and

Jinghong Zhao^1#^

^1^Department of Nephrology, Institute of Nephrology of Chongqing and Kidney Center of PLA, Xinqiao Hospital, Third Military Medical University, Chongqing, 400037, China

^2^Department of Nephrology, West China Hospital, Sichuan University, Chengdu 610041, China

^#^Corresponding author: Jinghong Zhao, Department of Nephrology, Institute of Nephrology of Chongqing and Kidney Center of PLA, Xinqiao Hospital, Third Military Medical University, Chongqing, 400037, China, Email: [zhaojh@tmmu.edu.cn](mailto:zhaojh@tmmu.edu.cn).

Supplementary figure 1. The quality of the including studies performed by QUDAS II.


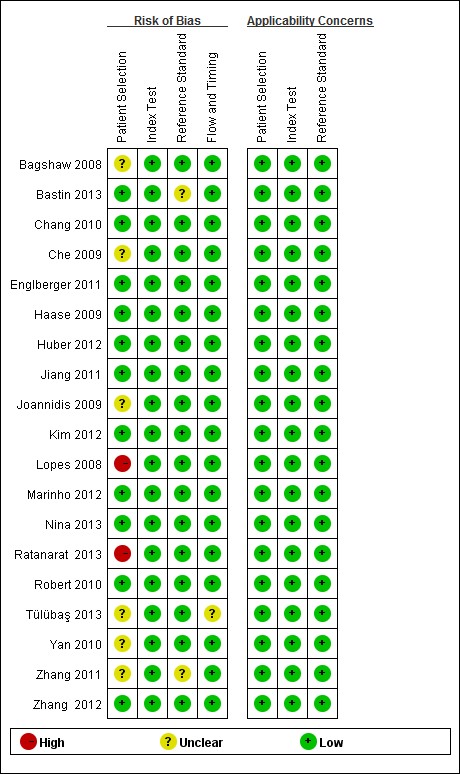


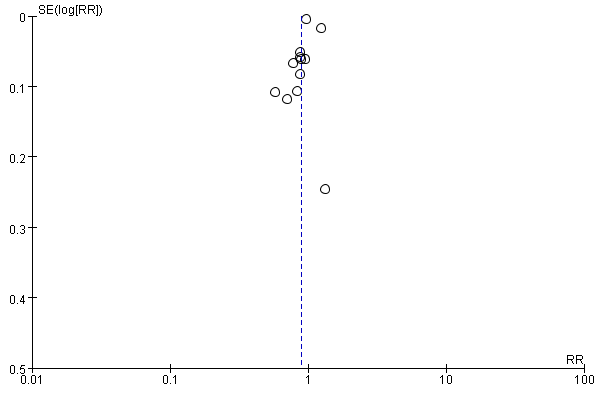
Supplementary figure 2. The funnel plot of the incidence of AKI in ICU patients


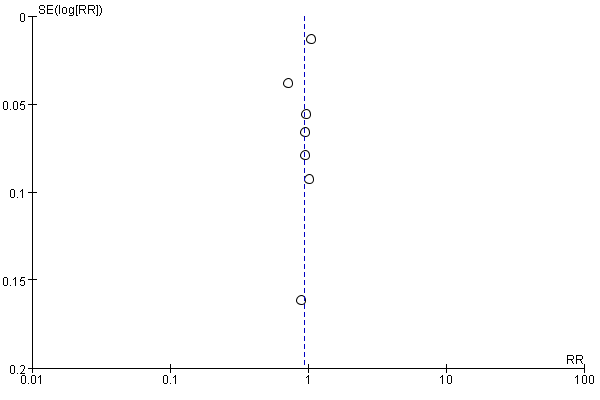
Supplementary figure 3. The funnel plot of the incidence of AKI in cardiac patients
